# A Universal Decoupled Training Framework for Human Parsing

**DOI:** 10.3390/s22165964

**Published:** 2022-08-09

**Authors:** Yang Li, Huahong Zuo, Ping Han

**Affiliations:** 1 School of Information Engineering, Wuhan University of Technology, Wuhan 430070, China; 2 Wuhan Chuyan Information Technology Co., Ltd., Wuhan 430030, China

**Keywords:** pixel resampling, long-tailed distribution, human parsing, semantic segmentation

## Abstract

Human parsing is an important technology in human–robot interaction systems. At present, the distribution of multi-category human parsing datasets is unbalanced, and the samples present a long-tailed distribution, which directly affects the performance of human parsing. Meanwhile, the similarity between different categories leads the model to predict false parsing results. To solve the above problems, a general decoupled training framework called Decoupled Training framework based on Pixel Resampling (DTPR) was proposed to solve the long-tailed distribution, and a new sampling method named Pixel Resampling based on Accuracy distribution (PRA) for semantic segmentation was also proposed and applied to this decoupled training framework. The framework divides the training process into two phases, the first phase is to improve the model feature extraction ability, and the second phase is to improve the performance of the model on tail categories. The training framework was evaluated in MHPv2.0 and LIP datasets, and tested in both high-precision and real-time SOTA models. The MPA metric of model trained by DTPR in above two datasets increased by more than 6%, and the mIoU metric increased by more than 1% without changing the model structure.

## 1. Introduction

A human–robot interaction system is the mutual transmission and exchange of information between humans and robots in a certain way, so as to cooperate to complete specific tasks. With the vigorous development of computer vision technology, camera sensors have become the mainstream medium for robots to obtain human information in modern human–robot interaction systems, and there is a large amount of image information that can be used as the carrier of human–robot interaction, such as gesture, facial expression, and body posture. With the diversified development of deep learning, computer vision has extended various types of tasks to adapt human–robot interaction in different scenarios. For example, R-CNN [1,2], YOLO [3], and SSD [4] implement object detection, which allows the robot to identify the specified person and object from the picture. Openpose [5] allows the robot to estimate the position of the skeletal joint points of the person in the image. To further utilize the human-related information in images, the human parsing task is proposed. Human parsing is a semantic segmentation task, which aims to identify human images pixel by pixel, and assign each pixel to a corresponding category, such as hair, arms, shirts, etc. At the end, these pixels come together to form a human parsing result. Human parsing helps to understand the semantic information of various parts of the human body in the image, and this information is very critical in character status analysis and character information analysis, which promotes the intelligence of human–robot interaction. With the great success of the fully convolutional neural network proposed by Long [6] in semantic segmentation, various excellent human parsing frameworks based on the FCN encoder–decoder paradigm emerged. For example, [7] proposed JPPNet using human key points, and [8] designed a CE2P human parsing framework using human edge information.

Although the human parsing task has been greatly developed, the current parsing tasks are only limited to rough category classification. The current large human parsing datasets such as ATR [9,10], LIP [11], CIHP [12], have great advantages in labeling quantity and quality. However, there is a slight lack in labeling categories. For example, the classification of clothing is limited to the rough classification of upper clothes, coat, and pants. In order to make machine vision understand the state information of the target person in the image better, it is necessary to refine the human parsing. The Fashionista dataset [13] contains 56 categories, and the training set contains 456 images, and the test set contains 229 images. The MHPv2.0 dataset [14] contains 25,403 images, and it has 59 annotation categories. Compared with the Fashionista dataset, the MHPv2.0 dataset provides a larger number of samples, which makes the model more robust. The increase in the number of categories also allows the model to learn more category information, mine, and understand the content in the image better. However, this brings corresponding problems: as the number of labeled categories increases, the distribution of large dataset is no longer balanced, which forms a long-tailed distribution. Moreover, as the labeled categories are gradually refined, the degree of imbalance in the dataset become more serious.

Aiming at the above problems, this paper proposes the Decoupled Training framework based on Pixel Resampling, which is a training framework designed for the long-tailed distribution of datasets in the semantic segmentation. The entire training framework is divided into two stages. The first stage uses the original dataset for normal training, focusing on training the encoder part of the model; the second stage freezes the network parameters of the encoder, focusing on training the decoder part of the model, and adopts the pixel resampling method to suppress the influence of data imbalance. Different from the traditional resampling, the pixel resampling proposed in this paper shifts the sampling object from the whole image to the pixels in the area where the category is located in the image. On this basis, we achieved the purpose of balanced sampling by changing the sampling probability corresponding to each category of pixels, which relieves the impact of long-tailed data distribution on the semantic segmentation model. It is worth noting that the decoupling training framework is suitable for various types of semantic segmentation structures, whether it is an encoder–decoder structure model such as PSPNet [15] and DeepLab [16,17,18] which have high segmentation accuracy, or a multi-path segmentation model such as BiSeNet [19,20] and DDRNet [21] that focus on segmentation speed, the training framework proposed in this paper can reduce the impact of dataset imbalance on the model. The experimental results show that this method can effectively solve the problem that the dataset follows a long-tailed distribution. This new detection work enjoys the following advantages:

1. A pixel resampling method in the field of semantic segmentation is proposed, which facilitates the realization of balanced sampling in semantic segmentation;
2. Based on the pixel-oriented resampling method, a decoupling training framework for semantic segmentation tasks is proposed, which can be applied to various human parsing models without changing the structure of the model. The training framework not only retains the powerful feature extraction ability of the model, but also alleviates the problem of the degradation of segmentation accuracy due to imbalanced dataset, which can additionally improve the segmentation performance of the model.

## 2. Related Work

### 2.1. Human Parsing

Human parsing is a fine-grained semantic segmentation task, and this research direction has received extensive attention due to its application potential in fields such as behavior recognition, virtual fitting, and virtual reality. The popular method is multi-task fusion, which concurrently takes human parsing and other similar tasks as the optimization target of the model. The authors of [11,22,23] optimized the effect of human parsing by integrating the human joint point prediction task. In [8], edge information was used as a sub-task of human parsing, and the accuracy of human parsing was improved by optimizing the model’s detection of edges. In addition to single-person human parsing, [14,24,25] studied human parsing tasks in multi-person scenarios. The authors of [26,27] applied joint parsing methods to parse images. The authors of [28] designed multiple parsers to perform clothing parsing using the similarity between images. The authors of [29] turned the attention from the network structure to the data labels, and found that the noise of the labels in the dataset has a great impact on the analytical ability of the model; it designs the SCHP to put the real labels into the model for iterative optimization, forming a more robust labels and models, which in turn improve model accuracy. However, the above work does not take into account the imbalance of the dataset, and with the refinement of the dataset annotation granularity the dataset tends to be more long-tailed distribution, and the analysis accuracy of the network is greatly reduced. Therefore, solving the data imbalance can further improve the model performance.

### 2.2. Long-Tailed Distribution

A dataset with a long-tailed distribution causes the model to overfit the head categories that account for the majority of samples, and underfit the tail categories that account for a small number of samples, which leads to a sharp drop in the accuracy rate. In the human parsing task, as the number of categories increases, the effect of the long-tailed distribution is particularly pronounced.

At present, there are three main methods to alleviate the long-tailed distribution. The first method is to make the number of each category as consistent as possible through resampling, under sample the category with more samples, and over sample the category with less samples to achieve the purpose of balanced sampling. The second method is to change the impact of different categories of data on the model by altering the loss function. For example, the loss function designed by [30] provides different weights to the corresponding categories according to the number of samples in different categories, thereby improving the accuracy of the category in the tail. The Focal Loss proposed by [31] increases the weight of hard samples and reduces the weight of easy samples to enhance the generalization ability of the model across all categories. The third method is transfer learning, Liu [27] transferred the deep features learned by the model from the category with a large number of head samples to the category with a small number of tail samples, which helps the model build a more complete feature space on the tail category, thus improving the accuracy of the tail category.

In the field of semantic segmentation, the common method to solve the problem of long-tailed distribution is to change the form of the loss function. For example, Seesaw Loss [32] alleviates the problem of the imbalance of positive and negative sample gradients in the tail category by weakening the negative gradient of the tail category. Loss Max-Pooling [33] reweights the loss value adaptively by designing a pixel weight function. In [34,35], it was found that a better solution to the classification task of long-tailed distribution is to decouple the training of the feature extraction network and the classifier. Firstly, the feature extraction network is trained through the cross-entropy function and the original data distribution. Then, the classifier behind the feature extraction network is trained through the balanced sampling data. In semantic segmentation tasks involving long-tailed distributed data, balanced sampling in the second stage of training is essential if this training paradigm is adopted. However, unlike the image classification task in which a picture represents a category, a picture in the semantic segmentation task has multiple categories, and there is a coupling relationship among categories. For example, the pictures containing tail categories such as jewelry and clothing of specific styles also contain head categories represented by faces and limbs, it is difficult to balance the frequency of all categories by resampling the entire image, so it is a huge challenge to achieve balanced sampling in semantic segmentation tasks.

To sum, decoupling training is an effective method to solve the problem of long-tailed distribution in view of the adverse effect of long-tailed distribution on human parsing caused by the refinement of dataset annotation. There is no better method for equalizing sampling of segmented objects in the field. Therefore, this paper proposes a pixel-based resampling method, and based on this, a general decoupled training framework for the human parsing model was constructed to solve the problem of long-tailed distribution of the dataset.

## 3. Methods

### 3.1. Overview

The essence of semantic segmentation is a pixel-level classification task. The structure of the segmentation network can be abstracted as an encoder–decoder structure. The input image extracts the low-level features through the encoder, such as color, texture, and edge, and then downsampling generates high-level semantic features. The decoder fuses and analyzes the high-level semantic feature information to infer the category of the pixel, and finally restores the resolution of the input image through upsampling.

**Figure 1:**
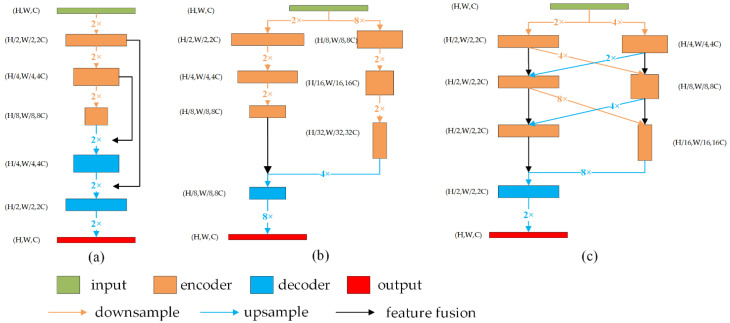
Different semantic segmentation network structures. We adopt direct addition as the feature fusion. (**a**) Typical U-shaped encoder–decoder structure represented by U-net [36]; (**b**) bilateral network structure represented by BiSeNet; (**c**) multi-resolution parallel network architecture represented by HRNet [37].

In this paper, the model was decoupled into two parts: encoder network and decoder network. Large-scale networks represented by PSPNet and DeepLab usually use the network structure of Figure 1a to integrate low-level features to improve segmentation details. The downsampling part constitutes the encoder part, and the upsampling part constitutes the decoder part. The definition of the encoder and the decoder is very clear. The real-time semantic segmentation network represented by BiSeNet is shown in Figure 1b. In order to reduce the amount of computation and delay, the decoder structure including upsampling is discarded. For the multi-scale parallel networks represented by HRNet and DDRNet, as shown in Figure 1c, the features of different scales have an upsampling decoder structure in the feature fusion stage, so the encoder and decoder cannot be clearly distinguished. However, a clear definition of decoder is necessary for decoupling the training framework. In order to improve the universality of the decoupling training framework proposed in this paper and make it applicable to most semantic segmentation networks, for the model structure shown in Figure 1b,c, this paper does not include these upsampling structures in the decoder set, but regards the last classification layer of the network as a special decoder.

**Figure 2:**
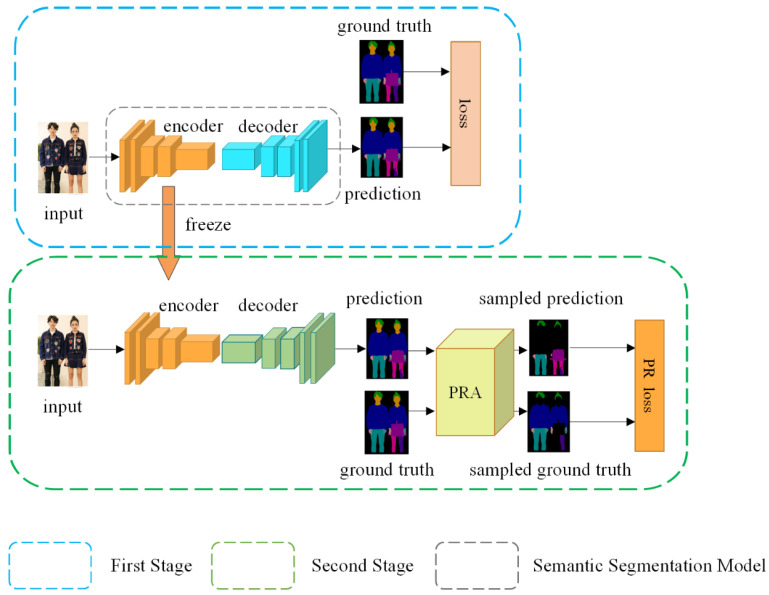
The structure of DTPR. The training process is divided into First Stage and Second Stage, and the frozen encoder in Second Stage comes from First Stage.

Figure 2 shows a schematic diagram of the Decoupling Training framework based on Pixel Resampling (DTPR). In the first stage, the model is trained according to the normal distribution of the dataset without the intervention of resampling. After training, the encoder part of the model is frozen and applied to the second stage. In the second stage, the image is sent into the model to obtain the model prediction, and then the prediction and ground truth are processed by PRA to generate the sampled prediction and sampled ground truth, and finally the loss function of the model is calculated using the sampled prediction and the sampled ground truth to optimize the model. It should be noted that the resampling in this paper occurs in the predicted graphs and the real labels, rather than the input images.

### 3.2. Pixel Resampling

Balanced resampling is an effective method to solve the problem of long-tailed distribution, but there is a coupling relationship among categories in semantic segmentation. Some categories, such as left and right hands, often appear in pairs, and it is difficult to achieve balanced sampling at the image level. Therefore, the sampling target is transferred from the entire image to the pixels of the specific category area in the image, and we call it pixel resampling. Only the sampled pixel area is included in the loss function to calculate the loss value.

(1) $$L_{pr}=\sum \left({\hat{y}}_{s},y_{s}\right)$$

where $\hat{y_{s}}$ is the sampled parsing results and $y_{s}$ represents the sampled ground truths, L is the loss function and $L_{pr}$ represents the loss function after pixel resampling.

Each category has a corresponding sampling probability. The sampling probability of a category determines the sampling frequency of the category. A high sampling probability indicates oversampling of the category, and a low sampling probability indicates undersampling of the category. If the data distribution of the dataset $X=x_{i},\;i\in 1,2,\ldots ,n$ where *n* is the number of the categories in dataset, the sampling probability distribution of each category $S=s_{i},\;i\in 1,2,\ldots ,n$, and the sampled data distribution $Y=y_{i},\;i\in 1,2,\ldots ,n$, and the equation can be provided as below:

(2) Y=X×S

Uniform distribution can effectively alleviate the problem of sample imbalance caused by long-tailed distribution data, so it is expected that the data distribution after sampling conforms to uniform distribution, so there is $y_{i}=\eta ,\forall i\in 1,2,\ldots ,n$, where η is a constant, then the corresponding sampling probability distribution is:

(3) $$s_{i}=\frac{y_{i}}{x_{i}}=\;\frac{\eta }{x_{i}}$$

In order to obtain the sampling probability $s_{i}$ of each category in Equation (3), it is necessary to count the data distribution X of the entire training dataset. We propose the Pixel Resampling based on accuracy distribution in this paper. The sampling method replaces the real data distribution X in the dataset with the Class Pixel Accuracy (CPA) of each category in the early stage of the model and obtains the sampling probability distribution S based on this, so as to determine the sampling method of each category pixel. CPA is defined as follows:

(4) $$\mathrm{CPA}\left(i\right)=\frac{\hat{Y_{i}}}{Y_{i}}$$

where $\hat{Y_{i}}$ is the number of pixels in the sample image that are correctly classified as the *i*-th class, and $Y_{i}$ is the number of pixels predicted by the model as the *i*-th class. The reason why the model adopts the CPA in the early training period instead of the late training period is that the accuracy rate in the late training period gradually loses the correlation with the samples due to the strong fitting ability of the neural network. We take the MHPv2.0 training dataset containing 59 categories as an example.

**Figure 3:**
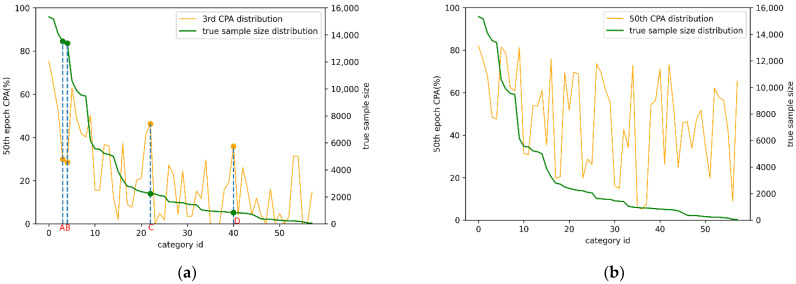
Comparison of Class Pixel Accuracy (CPA) distribution of different epochs and real data distribution in MHPv2.0 dataset. (**a**) The green curve represents the sample size distribution in MHPv2.0 dataset, and the yellow curve represents the CPA of each category. (**b**) We cannot find the correlation between the CPA distribution of 50th epoch and the correct rate distribution.

In Figure 3, the green curve is the distribution of the real samples in the dataset, and the yellow curve is the CPA distribution of different epochs. It can be seen that the CPA distribution of the third epoch is roughly in line with the distribution of the actual samples, however in the CPA distribution of the 50th epoch, the correct rate of many tail categories has been greatly improved, which is due to the powerful fitting ability of the neural network. After many iterations, the network learned the appropriate feature expression, so that the CPA distribution gradually deviates from the real sample size distribution. Therefore, the CPA distribution at the early stage of training can better replace the real samples distribution of the dataset.

It is worth noting that the CPA distribution of the third epoch in Figure 3a has a negative correlation between the correct rate of some categories and the real samples. For example, A, B, C, and D represent the left hand, right hand, dress, and ball. Although C and D belong to the tail category with a small number of samples, the corresponding CPA is very high. In contrast, A and B belong to the head category with a large number of samples, but the CPA is very low. The reason is that the similarity of the left hand and right hand categories makes it more difficult to identify these head categories, which in turn causes the CPA to be lower than expected. Categories such as dresses and balls have a regular shape and single style, which makes the neural network easier to fit, they lead to a high corresponding CPA. In view of the above phenomenon, it is necessary to provide more samples for the network to learn and recognize the differences between the head categories that are difficult to recognize, rather than just depending on the initial number of samples to determine whether the category is oversampled or undersampled. In the field of image classification, the distribution of balanced sampling is only determined by the number of samples. If the method is transferred to semantic segmentation, we call it Pixel Resampling based on Number distribution (PRN) in this paper, which leads to the under sampling of similar head categories that need to be oversampled, such as the left hand and right hand categories represented by the two points A and B mentioned above. The CPA distribution is the result of the model being affected by many factors such as the number of samples and the difficulty of classification. Therefore, the CPA distribution can more comprehensively describe the distribution of the entire dataset. Furthermore, this paper proposes the PRA, which avoids the shortcomings of PRN.

**Figure 4:**
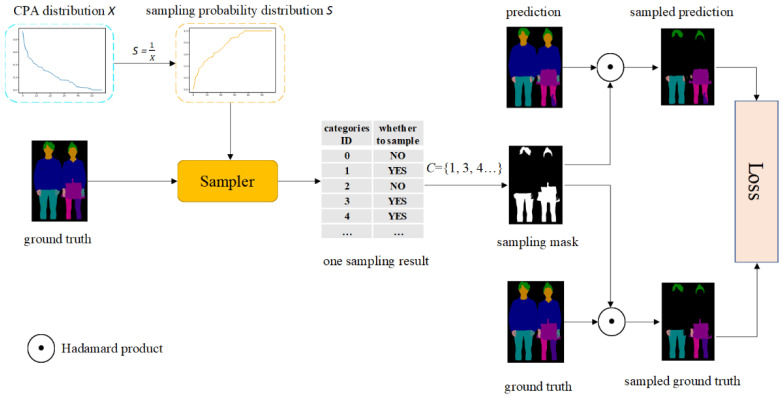
The structure of PRA. First, we calculate the sampling frequency distribution according to the CPA distribution, and the sampler samples the categories in the real label according to the sampling frequency distribution, and then generates the sampling mask. The sampling mask is used to perform a Hadamard product with the model prediction and the ground truth to obtain the sampled prediction and the sampled ground truth.

The structure of PRA is shown in Figure 4. The sampling probability of each category is obtained by calculating the CPA distribution of each category and Equation (3). The sampler samples the pixels in the image according to the sampling probability distribution of different categories. The categories sampled by the sampler in each ground truth are recorded, and we use these records to generate category set C to generate the sampling mask for this sampling process. In fact, the same ground truth may have different sampling mask in different batches. However, with the increase in batches, the sampling times of each category tend to the corresponding sampling probability. The definition of sampling mask is as follows:

(5) $$mask\left(x,y\right)=\left\{\begin{array}{c} 1{}^{\circ}G\left(x,y\right)\in C\\ 0{}^{\circ}G\left(x,y\right)\notin C \end{array}\right.$$

where $G\left(x,y\right)$ is the real tag, and then the prediction result and the real tag is point multiplied with the sampling mask to obtain the prediction result and the real tag after regional balanced sampling. Finally, they are sent to the loss function to calculate the loss value.

## 4. Experimental Result

### 4.1. Dataset

The proposed training framework is validated on two human parsing datasets, including the MHPv2.0 and LIP datasets. MHPv2.0 contains 25,403 elaborately annotated images with 58 fine-grained semantic category labels, involving 2–26 persons per image and captured in real-world scenes from various viewpoints, poses, occlusion, interactions, and background, of which 15,403 pictures are used as training sets, and 5000 pictures are used as verification sets. Moreover, in the experiment, different instances of the same category in the image are combined into a whole as the label of the semantic segmentation task. LIP is the largest human parsing dataset, which contains 50,462 images with elaborated pixel-wise annotations with 19 semantic human part labels. LIP is divided into 30,462 images for train set, 10,000 images for validation set and 10,000 for test set.

### 4.2. Evaluation Protocols

We mainly use three standard metrics, including pixel accuracy (PA), mean class pixel accuracy (MPA), and mean intersection over union (mIoU). Pixel accuracy reflects the proportion of correctly predicted pixels to total pixels:

(6) $$\mathrm{PA}=\frac{{\hat{Y}}_{correct}}{Y_{total}}$$

where ${\hat{Y}}_{correct}$ is the correctly predicted area, $Y_{total}$ represents the entire predicted area.

The CPA in Equation (4) is a measure of the recognition accuracy of a single category, and the MPA reflects the comprehensive recognition ability of the model for all categories:

(7) $$\mathrm{MPA}=\frac{\sum_{i=1}^{n}\mathrm{CPA}\left(i\right)}{n}$$

The mIoU is the main metric to generally judge the overall parsing performance of the method:

(8) $$\mathrm{mIoU}=\frac{\sum_{i=1}^{n}\mathrm{IoU}\left(i\right)}{n}$$

(9) $$\mathrm{IoU}\left(i\right)=\frac{p_{ii}}{\sum_{j=0}^{n-1}p_{ij}+\sum_{j=0}^{n-1}p_{ji}-p_{ii}}$$

We assume a total of n classes from 0 to *n* − 1 including a background, and $p_{ij}$ is the amount of pixels of class *i* inferred to belong to class *j*. In other words, $p_{ii}$ represents the true positives, while $p_{ij}$ and $p_{ji}$ are false positives and false negatives, respectively.

### 4.3. Implementation Details

In this experiment, Intel Core i9-10900K CPU@3.70 GHz, NVIDIA GPU 3090, 64 GB RAM is selected as the hardware platform, and the operating system is Ubuntu18.04 which is based on Debian. The high precision segmentation network chose ResNet-50 [38] as the encoder network and used the pretrained weights from ImageNet [3]. Considering the diversity of the image size of the dataset, we used a resolution of 400 × 400 as the input of the model, and the batch size used in the training process was 16, the optimizer adopted SGD, the initial learning rate was set to 0.01, and the learning rate strategy adopted CosineAnnealing. The image enhancement methods used during training include image rotation (−10° to 10°) and image cropping.

### 4.4. Experimental Results and Analysis

#### 4.4.1. Comparison of Pixel Sampling Methods

In order to explore the difference between the effects of PRA and PRN, PSPNet was used as the semantic segmentation model in the experiment. The experiment was divided into three groups. The first group did not use the decoupling training framework to train 100 epochs, and the second group used the decoupling training framework and adopt the PSA in the second stage. The third group used the decoupling training framework and adopt the PSN in the second stage. For the fairness of the experiment, the number of epochs for the three groups of training is the same, 100 epochs for non-decoupling training, 70 epochs for the first stage, and 30 epochs for the second stage of decoupling training.

From the results in Table 1, we can see that using PRA achieves more gains in the evaluation metrics of PA and mIoU, while using PRN achieves more gains in MPA; however, the mIoU can better reflect the segmentation performance of the model. In this metric, PRA has an additional 0.8% improvement compared with PRN.

**Figure 5:**
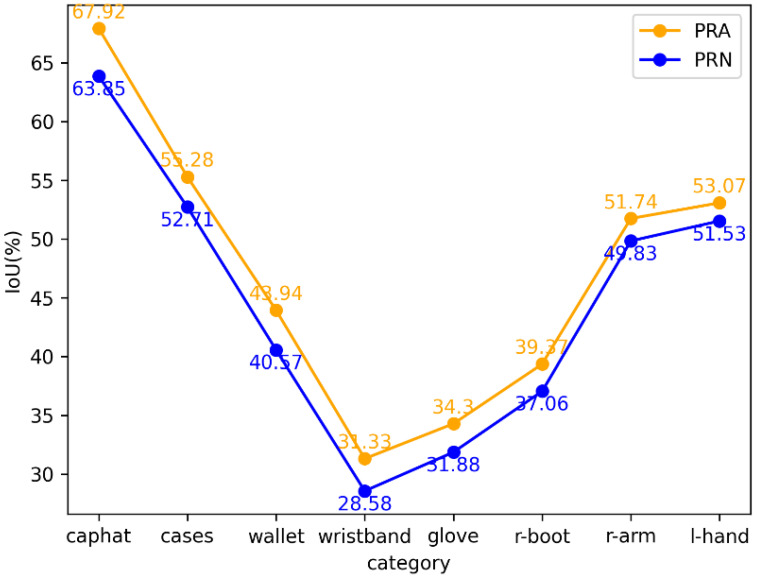
Comparison of IoU between PRA and PRN in some categories on the MHPv2.0 validation set.

It can be seen from Figure 5 several categories in which PRA is more improved than PRN, all have the characteristics of being difficult to classify, such as caphat, cases, wallet, wristband, and glove, which have many styles and small volumes, and the right-boot, right arm, and right hand have symmetry and are easily confused with the corresponding mirror categories. For the head categories with a large number of samples, PRN selects undersampling according to the number of samples. It can be seen from Figure 3a that the CPA of these difficult-to-classify head categories are at a very low level in the early stage of training, so PRA oversamples these categories, allowing the model to use more samples to learn more robust features.

#### 4.4.2. Performance on the MHPv2.0 Dataset

##### High Accuracy Model

In order to ensure the fairness of the experiment, the contrast group without PRA normally trained 100 epochs, and the experimental group with PRA continued to train 30 epochs with the 70th epochs training model of the contrast group. As can be seen from Table 2, for each semantic segmentation model trained by DTPR, three evaluation metrics improved, especially the MPA metric, which increased by more than 6% compared with the previous model. Moreover, the performance about other methods which are designed for the imbalanced dataset are in Table 3.

**Figure 6:**
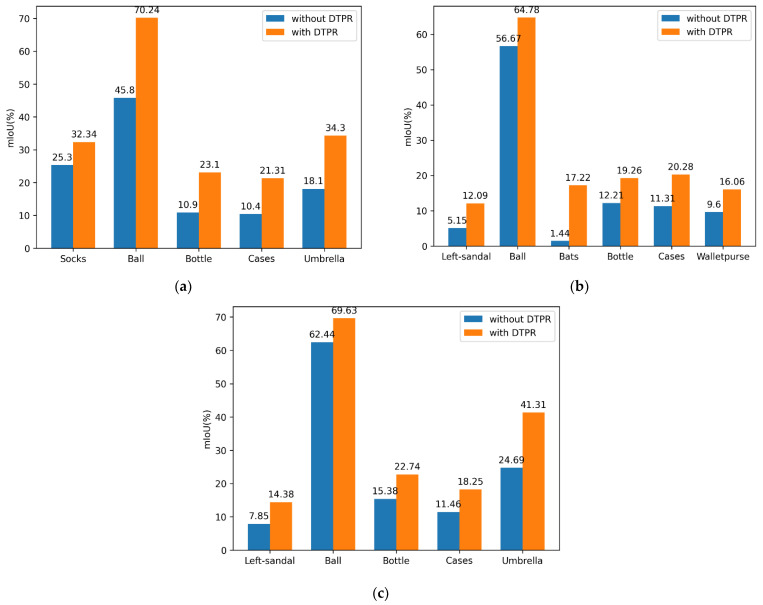
The mIoU performance with different models on some categories. (**a**) PSPNet is used as the semantic segmentation model. (**b**) Deeplab3+ is used as the semantic segmentation model. (**c**) DANet is used as the semantic segmentation model.

We extracted the categories that were improved more after DTPR training, and the results are shown in Figure 6.

Owing to pixel-balanced sampling, which allows the model to learn all class features in a balanced manner, and imbalanced datasets are more balanced during model training, which can greatly improve the accuracy of tail categories. The reason why the mIoU metric is not as improved as the MPA metric is that the evaluation of mIoU is related to the number of samples, so mIoU mainly depends on the IoU of the head category. It can be seen that after pixel resampling, the improvement effect of IoU of the tail category is very significant. For example, the mIoU of ball in DeepLab increased by 8.11%, in PSPNet by 24.44%, and in DANet by 7.19%. In addition to the tail category there is also a great improvement on the hard-to-classify categories. For example, the symmetrical left-sandal improves 7.75% in PSPNet, and 6.53% in DANet.

**Figure 7:**
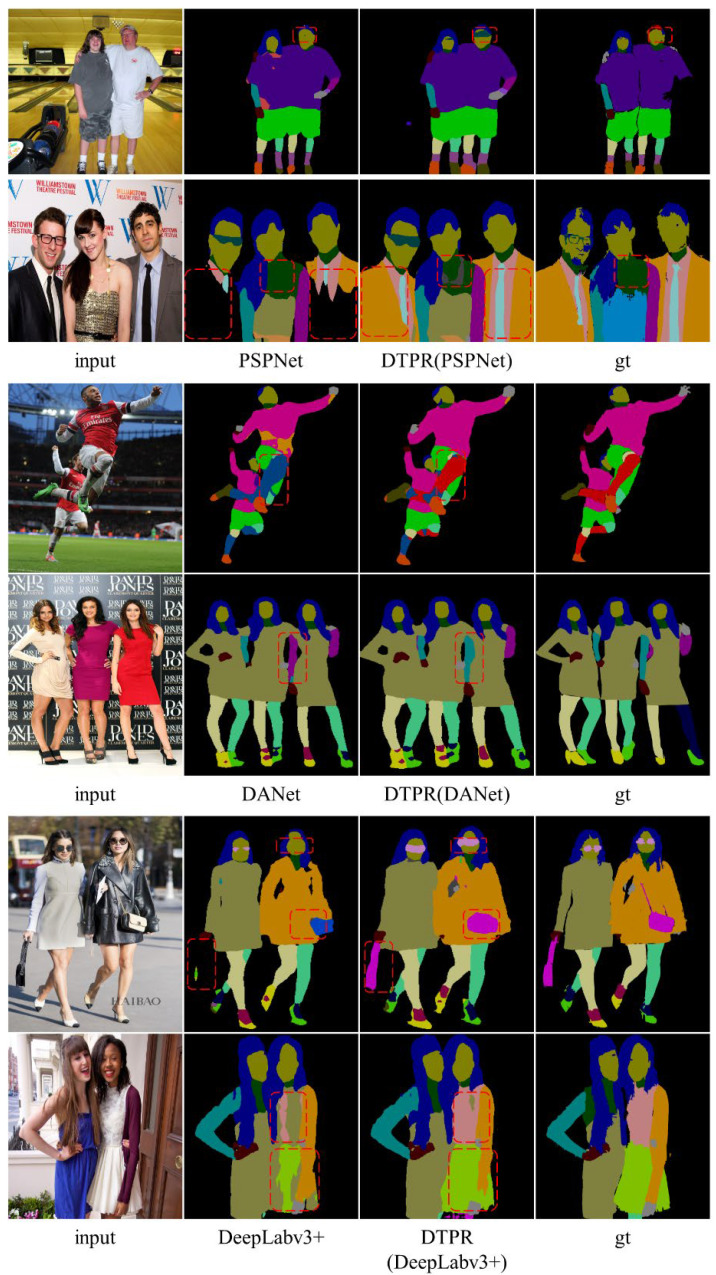
Visual comparison of human parsing results on the MHPv2.0 validation set.

Based on the MHPv2.0 dataset, this paper visually compares the effects before and after DTPR processing, as shown in Figure 7. It can be seen that the model after DTPR is more accurate in identifying uncommon categories such as jewelry (the second row). Due to the powerful feature extraction ability of the semantic segmentation network, the model has good recognition ability for small-volume items such as transparent glasses and occluded hats (the first row). These categories are not even marked by the ground truth labels. In addition, the model has a lower error rate for the categories that are difficult to recognize, such as the left and right arms (the fourth row).

##### Lightweight Model

For the real-time semantic segmentation model that pays more attention to speed, the decoupling training framework in this paper can also improve the recognition ability of the model without affecting the inference speed. This paper uses the three most advanced real-time semantic segmentation model: BiSeNetv2, STDC, and DDRNet to verify the validity of DTPR.

As can be seen from Table 4, for lightweight networks with weak feature extraction capabilities, DTPR significantly improves the performance of the model. In the evaluation metrics of MPA, BiSeNetv2, STDC, and DDRNet are improved by 8.32%, 6.72%, and 8.22%, respectively. In addition, gains of 2.58%, 1.54%, and 3.07% are obtained on the mIoU metric, respectively. Compared with the previously mentioned deep networks, these lightweight networks achieve greater performance through DTPR, which fully demonstrates that the data distribution of the dataset has a huge impact on the model.

#### 4.4.3. Performance on the LIP Dataset

##### High-Accuracy Model

For the LIP dataset, we used the LIP validation set as a test, and the results of the three evaluation metrics in the three SOTA model are shown in Table 5, and we also list the IoU metrics for each category in Table 6.

It can be seen from Table 5 that DTPR can account for the lack of network feature extraction ability to a certain extent. For example, in the hat category, the mIoU of DANet with the strongest feature extraction ability is 57.08%, and the mIoU of DeepLabv3+ with the weakest extraction ability is 55.08%. After DPTRS training, the recognized mIoU of both networks is around 57%. In addition, on the whole, the model trained by DTPR has more obvious improvement in tail categories and confusing categories, such as glove, scarf, and left shoe.

**Figure 8:**
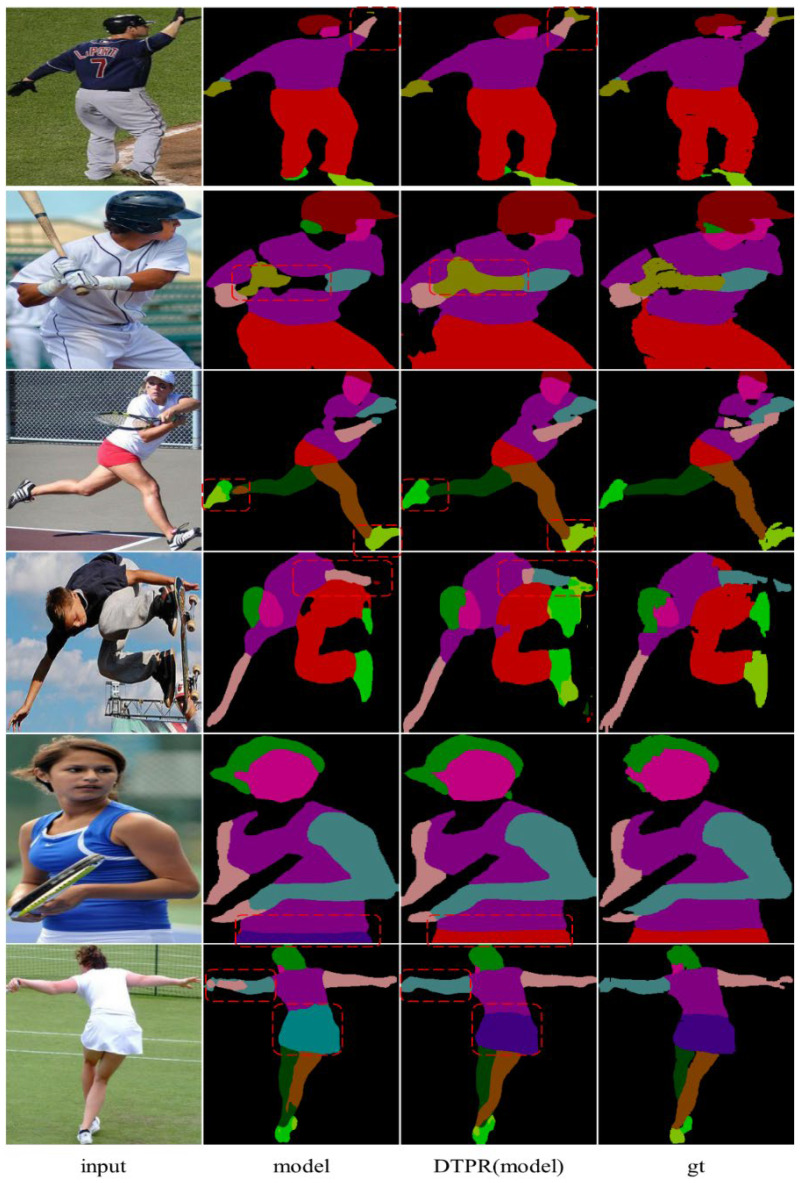
Visual comparison of human parsing results on the LIP validation set.

Six representative images chosen from the LIP validation dataset are shown in Figure 8. The first two lines show that the model trained by DTPR can recognize the tail category more precisely, such as the gloves in the picture. In addition, the DTPR-trained model achieves better parsing results than the original model in both mirror categories (left and right arms, left and right shoes) and hard-to-classify samples (shorts and skirts).

##### Lightweight Model

After the lightweight network is trained with DTPR, the results are very impressive, which are shown in Table 7 and Table 8. Not only was the MPA greatly improved, but also the mIoU and PA metrics were greatly improved. It is worth noting that the performance of the STDC with only 9 M parameters after DTPR training is close to the DeepLabv3+ with 59 M parameters, which shows that the lightweight network has a lot of potential to be tapped, and the reasonable distribution of the dataset is an effective means to improve lightweight model performance.

## 5. Conclusions

Aiming at the problem of low human parsing accuracy caused by the long-tailed distribution of data in multi-category datasets, this paper proposes a decoupling training framework in the field of semantic segmentation. In order to achieve balanced training of the second-stage classifier, this paper proposes a method based on pixel resampling, and the resampling effect based on the distribution of accuracy rate and the distribution of sample number is compared. In addition, this paper uses the MHPv2.0 and LIP datasets as benchmarks, and conducts a large number of comparative experiments between the SOTA model that focuses on accuracy and the SOTA model that focuses on speed. The experimental results fully demonstrate the effectiveness of the DTPR proposed in this paper. However, in order to improve the universality of DTPR, for the network with a typical encoder–decoder structure, this paper only placed its last classification layer as the last decoder layer in the second stage of training, which undoubtedly limits the performance of the model. Hence, how to define the decoder layer more effectively needs to be further explored in future work.

## Figures and Tables

**Table 1:** Comparison of pixel sampling method based on different data distributions.

Method	PA	MPA	mIoU
PSPNet	71.84	45.18	35.92
PSPNet+PRN	72.02	53.06	36.77
PSPNet+PRA	72.69	52.22	37.57

**Table 2:** Comparison of three SOTA models on the MHPv2.0 validation set.

Method	PA		MPA		mIoU	
	No DTPR	With DTPR	No DTPR	With DTPR	No DTPR	With DTPR
PSPNet	71.84	72.69	45.18	52.22	35.92	37.57
DeepLabv3+	71.67	72.42	44.56	51.19	34.98	36.76
DANet	72.06	72.63	45.92	52.22	36.51	37.70

**Table 3:** Comparison of three method for imbalanced dataset.

Method	PA	MPA	mIoU
Focal Loss [31]	71.51	44.89	35.57
CB Loss [30]	72.02	48.52	36.77
Our Method	72.69	52.22	37.57

**Table 4:** Comparison of the real-time model on the MHPv2.0 validation set.

Method	PA		MPA		mIoU	
	NoDTPR	With DTPR	No DTPR	With DTPR	No DTPR	With DTPR
BiSeNetv2	59.14	59.88	25.96	34.28	20.28	22.86
STDC	63.47	63.89	29.77	36.49	23.34	24.88
DDRNet	61.91	62.26	25.74	33.96	19.96	23.03

**Table 5:** Comparison of the SOTA model on the LIP validation set.

Method	PA		MPA		mIoU	
	No DTPR	With DTPR	No DTPR	With DTPR	No DTPR	With DTPR
PSPNet	70.80	74.95	49.58	54.20	42.23	44.22
DeepLabv3+	70.57	73.70	44.56	51.19	34.98	36.76
DANet	71.26	74.10	50.39	54.11	42.86	44.29

**Table 6:** Comparison of the SOTA model on the LIP validation set.

	PSPNet	DTPR (PSPNet)	DeepLabv3+	DTPR (DeepLabv3+)	DANet	DTPR (DANet)
Hat	55.08	57.33	52.02	57.55	57.08	57.52
Hair	66.41	69.58	62.75	68.64	67.33	69.23
Glove	34.14	41.07	33.60	40.70	36.85	40.68
S-glasses	31.49	32.16	26.06	32.01	31.17	33.97
Up-Cloth	65.59	66.88	64.32	66.96	65.77	66.38
Dress	20.77	21.66	20.37	23.16	18.66	26.44
Coat	51.41	51.69	49.34	51.54	50.92	51.79
Socks	40.43	41.67	37.68	38.88	41.23	41.91
Pants	71.05	72.42	72.70	73.84	71.27	73.17
Jumpsuits	26.96	27.88	27.94	27.93	28.14	27.42
Scarf	12.88	16.17	13.89	17.95	15.83	15.06
Skirt	15.59	17.73	14.64	17.72	16.09	15.30
Face	78.03	80.13	74.10	77.50	79.07	76.70
L arm	57.84	60.61	59.73	60.73	59.15	60.43
R arm	61.43	63.73	61.97	62.47	61.97	62.99
L leg	47.93	49.37	48.36	51.59	47.90	51.75
R leg	49.76	50.35	49.26	49.88	49.89	52.25
L shoe	29.27	32.61	38.03	33.19	29.67	32.01
R shoe	28.28	31.35	29.59	29.78	29.23	30.78
Avg	42.23	44.22	41.32	44.10	42.86	44.29

**Table 7:** Comparison of the real-time model on the MHPv2.0 validation set.

Method	PA		MPA		mIoU	
	No DTPR	With DTPR	No DTPR	With DTPR	No DTPR	With DTPR
BiSeNetv2	63.30	59.37	42.63	36.17	32.10	29.88
STDC	69.24	65.00	48.25	43.21	38.28	35.53
DDRNet	68.42	64.75	47.41	40.32	36.48	33.80

**Table 8:** Comparison of the SOTA model on the LIP validation set.

	PSPNet	DTPR (PSPNet)	DeepLabv3+	DTPR (DeepLabv3+)	DANet	DTPR (DANet)
Hat	38.34	37.57	46.70	49.49	46.00	49.17
Hair	56.91	57.03	59.77	61.91	59.23	60.52
Glove	15.35	23.25	26.34	30.50	19.47	26.46
S-glasses	15.32	19.72	24.42	28.68	17.65	24.30
Up-Cloth	53.78	55.03	60.70	61.87	59.59	60.60
Dress	7.80	11.80	13.55	15.28	12.80	14.99
Coat	38.76	40.01	44.72	46.75	44.18	45.52
Socks	24.49	26.25	28.05	31.00	27.26	30.36
Pants	58.44	61.26	66.91	69.05	65.53	68.13
Jumpsuits	18.35	16.60	22.66	21.62	18.33	20.02
Scarf	4.03	5.21	5.07	7.69	6.35	8.02
Skirt	7.22	9.82	13.20	13.40	10.35	11.13
Face	71.20	72.85	74.61	77.17	73.12	75.71
L arm	45.13	48.54	52.84	54.95	49.76	52.72
R arm	49.26	52.32	54.66	58.36	53.40	56.30
L leg	33.34	34.28	43.20	43.68	37.26	40.55
R leg	32.67	35.24	41.72	44.27	40.08	41.66
L shoe	15.12	17.56	22.58	24.96	17.97	21.96
R shoe	12.14	17.69	21.49	25.06	17.68	21.58
Avg	29.88	32.10	35.53	38.28	33.80	36.48

## Data Availability

Not applicable.
